# Long-term outcome of percutaneous radiofrequency ablation for periportal hepatocellular carcinoma: tumor recurrence or progression, survival and clinical significance

**DOI:** 10.1186/s40644-021-00442-2

**Published:** 2022-01-04

**Authors:** Shoujin Cao, Tianshi Lyu, Zeyang Fan, Haitao Guan, Li Song, Xiaoqiang Tong, Jian Wang, Yinghua Zou

**Affiliations:** Department of Interventional and Vascular Surgery, Peking University First Hospital, No. 8 Xishiku Street, Xicheng District, Beijing, 100034 China

**Keywords:** Hepatocellular carcinoma (HCC), Periportal, Radiofrequency ablation (RFA), Tumor recurrence, Tumor progression, Therapeutic outcomes

## Abstract

**Background/aim:**

Recent studies have suggested that periportal location of percutaneous radiofrequency ablation (RFA) for hepatocellular carcinoma (HCC) is considered as one of the independent risk factors for local tumor progression (LTP). However, the long-term therapeutic outcomes of percutaneous RFA as the first-line therapy for single periportal HCCand corresponding impacts on tumor recurrence or progression are still unclear.

**Materials and methods:**

From February 2011 to October 2020, a total of 233 patients with single nodular HCC ≤ 5 cm who underwent RFA ± transarterial chemoembolization (TACE) as first-line therapy was enrolled and analyzed, including 56 patients in the periportal group and 177 patients in the nonperiportal group. The long-term therapeutic outcomes between the two groups were compared, risk factors of tumor recurrence or progression were evaluated.

**Results:**

The LTP rates at 1, 3, and 5 years were significantly higher in the periportal group than those in the nonperiportal group (15.7, 33.7, and 46.9% vs 6.0, 15.7, and 28.7%, respectively, *P* = 0.0067). The 1-, 3- and 5-year overall survival (OS) rates in the periportal group were significantly worse than those in the nonperiportal group (81.3, 65.1 and 42.9% vs 99.3, 90.4 and 78.1%, respectively, *P<*0.0001). In the subgroup of single HCC ≤ 3 cm, patients with periportal HCC showed significantly worse LTP *P* = 0.0006) and OS (*P*<0.0001) after RFA than patients with single nonperiportal HCC; The univariate and multivariate analyses revealed that tumor size, periportal HCC and AFP ≥ 400ug/ml were independent prognostic factors for tumor progression after RFA. Furthermore, patients with single periportal HCC had significantly higher risk for IDR(*P* = 0.0012), PVTT(*P*<0.0001) and extrahepatic recurrence(*P* = 0.0010) after RFA than those patients with single nonperiportal HCC. .

**Conclusion:**

The long-term therapeutic outcomes of RFA as the first-line therapy for single periportal HCC were worse than those for single nonperiportal HCC, an increased higher risk of tumor recurrence or progression after RFA was significantly associated with periportal HCC.

**Supplementary Information:**

The online version contains supplementary material available at 10.1186/s40644-021-00442-2.

## Introduction

Radiofrequency ablation (RFA) has been widely used for the treatment of hepatocellular carcinoma (HCC) during the last decade. As it is minimally invasive and potentially curative, RFA is currently considered the best option for patients with early HCC who are not candidates for surgical intervention [1, 2]. Unlike surgical intervention for HCC, the major obstacle of RFA is frequent local tumor progression (LTP) after ablation, which is directly associated with tumor recurrence. The 5-year LTP rates reported previously range from 3.2 to 27.0%, and the incidence of HCC recurrence reached 63.3% after a mean follow-up period of 38 months [3, 4]. Frequent HCC recurrence after RF ablation undoubtedly impairs long-term survival outcomes.

Currently, many risk factors for LTP have been confirmed in previous studies, such as tumor size, insufficient ablative margin, indistinct tumor margin, hypervascular HCC, and unfavorable location of HCC [3, 5–7]. Recent studies have suggested that periportal location is an important risk factor for LTP after RFA [8, 9]. In addition, several unusual tumor recurrence patterns closely associated with periportal HCC after RFA have been observed in multiple reports, such as intersegmental recurrence [10], aggressive intersegmental recurrence (AIR) [11], and rapid and scattered recurrence [9, 12], and most of these recurrence patterns tend to worsen prognosis. However, the risk of recurrence and prognosis after ablation of periportal HCC remain unclear compared to nonperiportal HCC. A retrospective comparative study reported previously suggested that the therapeutic outcomes of RFA for small (≤3 cm) perivascular HCC were equivalent to those for nonperivascular HCC [13]. But a recent study suggested that “periportal” but not “perivenous” should replace “perivascular” as a risk factor for LTP [8]. Another study found that periportal HCC was one of the risk factors for intrasegmental recurrence [10].

Therefore, further studies are warranted to evaluate the clinical outcomes, potential patterns of recurrence, and clinical significance of periportal HCC after RFA compared to nonperiportal HCC. The purpose of the study was to compare the long-term therapeutic outcomes of percutaneous RFA as the first-line therapy for single periportal HCC with nonperiportal HCC, potential risk factors of tumor recurrence or progression after RFA were evaluated.

## Materials and methods

### Patients

**Fig. 1:**
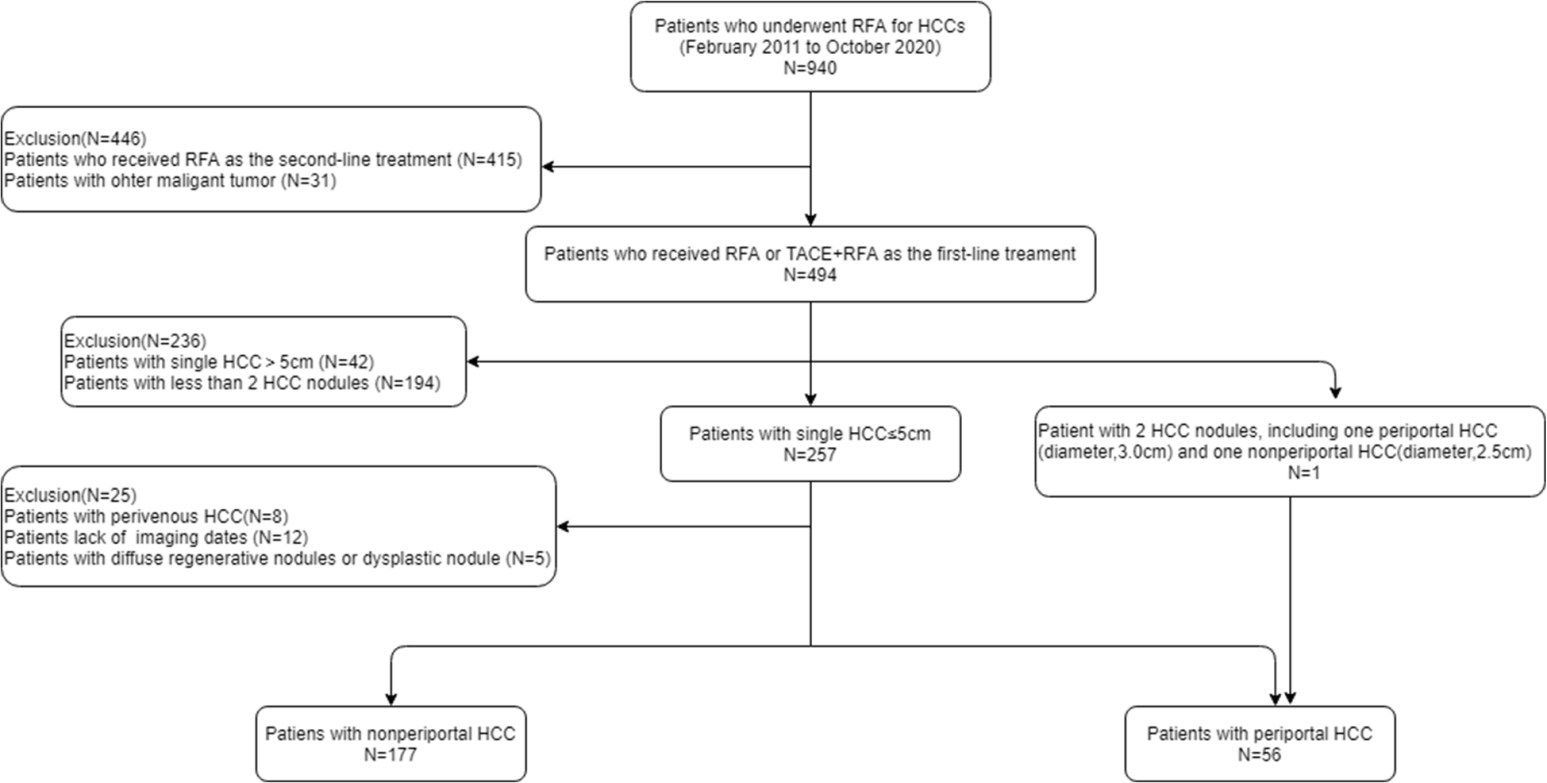
The flow diagram showing exclusion criteria in patients with hepatocellular carcinoma (HCC) who underwent radiofrequency ablation (RFA) or transarterial chemoembolization (TACE) combined with RFA (TACE+RFA)

We conducted a retrospective study of HCC patients who underwent RFA or RFA combined with transarterial chemoembolization (TACE-RFA) as the initial treatment in our institution from February 2011 to October 2020. This study was approved by our institutional review board. Written informed consent was obtained from all patients before each treatment. The inclusion criteria for this study included patient with the following characteristics: (a) BCLC stage A with a single HCC mass; (b) HCC ≤ 5.0 cm in diameter; and [3] one patient with 2 HCC nodules, including one periportal HCC (diameter, 3.0 cm) and one nonperiportal HCC (diameter, 2.5 cm) with curative ablation after RFA. The exclusion criteria for this study were as follows: (a) patients who had severe cirrhosis with diffuse regenerative nodules or dysplastic nodules; (b) patients with HBV or HCV who did not receive regular antiviral therapy; (c) patients who had other previous or concomitant malignancies; (d) patients with perivenous HCC; and (e) patients lacking imaging data. HCC patient diagnoses were all confirmed according to the histopathology or European Association for the Study of Liver/American Association for the Study of Liver Disease guidelines. Accordingly, 233 patients, including 56 patients with periportal HCC and 177 patients with nonperiportal HCC, were enrolled and analyzed in this study (Fig. 1).

### The procedures of RFA with or without TACE

TACE was performed by superselectively inserting microcatheters (Asahi Intecc Co., Ltd., Japan) into the tumor’s feeding arteries. Then, 10–30 mg of doxorubicin mixed with 5–10 mL of lipiodol (Guerbet, Villepinte, Seine-Saint-Denis, France) was injected slowly under continuous fluoroscopic guidance until blood flow in the tumor-feeding arteries ceased. The decision of whether to perform TACE before RF ablation was depended not only on tumor factors (tumor size, location, and poor conspicuity), but also on individual factors (such as patient wishes and economic affordability). In our center, we prefer TACE before ablation for tumors larger than 3 cm in diameter, with unfavorable ablation locations (such as subphrenic, and subphrenic HCC, adjacent to the major vessels, gallbladder, or gastrointestinal tract) and HCCs with poor conspicuity. The time interval between cTACE and RFA was usually within 4 weeks.

RFA procedures were performed under multi-imaging guidance, including ultrasonography (US) combined with computed tomography (CT) or US combined with cone-beam CT (CBCT). US was primarily used in tumor puncture and real-time monitoring of the ablation process, and CT/CBCT was used for the evaluation of the ablation zone. We used a RITA Medical Systems (RITA 1500X RF generator, RITA Medical Systems; AngioDynamics, Manchester, Ga) according to the manufacturer’s standard recommendations for radiofrequency power settings and ablation times. Two types of electrode needles (Starburst xl, Rita Medical System; Vascular Dynamics) were used for HCC, including a 2-cm expandable electrode and a 3-cm expandable electrode. Multiple adjustments of the location of the needle tip were performed to obtain a sufficient ablation zone, and the puncture tract was finally ablated during electrode retraction to prevent bleeding or tract seeding. All procedures were performed by the same team with extensive experience in interventional therapy in our center.

### Follow-up and treatment strategy for recurrent tumors

Contrast-enhanced multiphase CT or MR and alpha fetoprotein (AFP) were performed 1 month after each treatment. If no evidence of residual tumor or HCC recurrence was found, the follow-up was performed every 3 months until two years after RFA. Then, the interval of follow-up was adjusted to every 3–6 months according to the tumor stage or the risks for recurrence. If residual tumor or HCC recurrence was confirmed during the follow-up visits, optimal treatment was conducted according to the clinical practice guidelines of HCC, the multidisciplinary team for HCC management and the general condition of the patient, including ablation, surgical resection, liver transplantation, TACE, systemic therapy, radiation therapy or combination therapy. RFA alone or combined with TACE was preferably used to treat residual tumor, LTPs or new tumor foci in our center.

### Assessments and definitions

Periportal HCC was defined as the margin of HCC within 5 mm from the major portal vein (first to third branches of the portal vein). Nonperiportal HCC was defined as the margin of HCC beyond 5 mm from the major portal vein (first to third branches of the portal vein) and the hepatic veins (first to second branches of the hepatic vein) or inferior vena cava. Residual unablated HCC was defined as residual visible tumor observed at the 1-month follow-up after initial RFA. LTP was defined as the appearance of enhancing HCC foci at the edge of the ablation zone [14]; Intrahepatic distant recurrence (IDR) was defined as any new emerging tumor that occurred in the liver separate from the ablated zone; Tumor progression was defined as the appearance of any enhancing tumor foci in the liver or extrahepatic metastasis during the follow-up after ablation, including LTP, IDR, vascular invasion, extrahepatic metastasis.

### Statistical analysis

Continuous dates were compared using the t-test, and categorical dates were compared using the chi square test. Local tumor progression, disease-free survival, and overall survival were calculated using the Kaplan–Meier method, and differences between groups were compared using the log-rank test. The univariable and multivariable analysis of prognostic factors for local tumor progression and tumor progression were assessed using Cox proportional hazard models. Patients who underwent surgery or liver transplantation during follow-up were censored. All reported *P*-values are 2-sided, with *P* < 0.05 considered statistically significant. All statistical analyses were performed using the GraphPad Prism 8.0.1(244).

## Results

### Baseline characteristics of the patients

A total of 233 patients received RFA or TACE+RFA as an initial treatment for HCC, including 56 patients in the periportal group and 177 patients in the nonperiportal group. There were no significant differences between the periportal and nonperiportal groups with respect to mean age, sex ratio, tumor size, cirrhosis ratio, tumor etiology, Child–Pugh classification, serum AFP level, proportion of types of electrode needles or proportion of patients with TACE+RFA (Table 1).

**Table 1:** Baseline characteristics of the 233 patients who received radiofrequency ablation as the first-line option for hepatocellular carcinoma

Variable	Periportal HCC(*n* = 56)	Nonperiportal HCC (*n* = 177)	*P*-value
Age (years)	59.4 ± 11.8 (37 ~ 85)	59.5 ± 9.9 (33 ~ 88)	0.966
Sex, n (%)			
Male	43 (76.8%)	145 (81.9%)	
Female	13 (23.2%)	32 (18.1%)	
Tumor size (cm)			0.190
Mean ± standard deviation	2.9 ± 1.1 (0.9–5.0)	2.6 ± 1.1 (0.8–5.0)	
≤ 3.0 cm	35 (62.5%)	127 (71.8%)	
3.1–5.0 cm	21 (37.5%)	50 (28.2%)	
Types of electrode needles			0.107
2-cm expandable electrode	31 (55.4%)	119 (67.2%)	
3-cm expandable electrode	25 (44.6%)	58 (32.8%)	
Cirrhosis			0.579
Yes	39 (69.6%)	130 (73.4%)	
No	17 (30.4%)	47 (26.6%)	
Etiology of tumor			0.823
Hepatitis B virus	44 (78.6%)	135 (76.3%)	
Hepatitis C virus	6 (10.7%)	15 (8.5%)	
Other	2 (3.6%)	10 (5.6%)	
No	4 (7.1%)	17 (9.6%)	
Child–Pugh classification			0.700
A	49 (87.5%)	164 (92.7%)	
B	7 (12.5%)	13 (7.3%)	
Serum AFP (ng/ml)			0.38
Mean ± standard deviation	264.7 ± 517.1 (1.82 ~ 2449.3)	229.6 ± 991.7	
≤ 20	34 (60.7%)	(1.10 ~ 9346.9)	
>20 and<400	12 (21.4%)	106 (59.9%)	
≥ 400	10 (17.9%)	50 (28.2%)	
		21 (11.9%)	
TACE before RFA			0.821
Yes	37 (66.1%)	114 (64.4%)	
No	19 (33.9%)	63 (35.6%)	

### Technical success

The technical success rates after initial RFA were 91.1% (51/56) in the periportal group and 96.0% (170/177) in the nonperiportal group(*P* = 0.1662). Technical failure after initial RFA was attributed to mistargeting (1 patient in the periportal group and 2 patients in the nonperiportal group), residual unablated tumors (2 patients in the periportal group and 5 patients in the nonperiportal group), and rapid portal vein tumor thrombus after initial RFA (2 patients in the periportal group). Except for 2 patients in the periportal group who experienced portal vein invasion caused by unablated residual HCC after initial RFA, the other 8 patients immediately underwent an additional RFA session with complete ablation owing to primary technical failure.

### Tumor progression or recurrence, prognostic factors of LTP, and salvage treatments

**Fig. 2:**
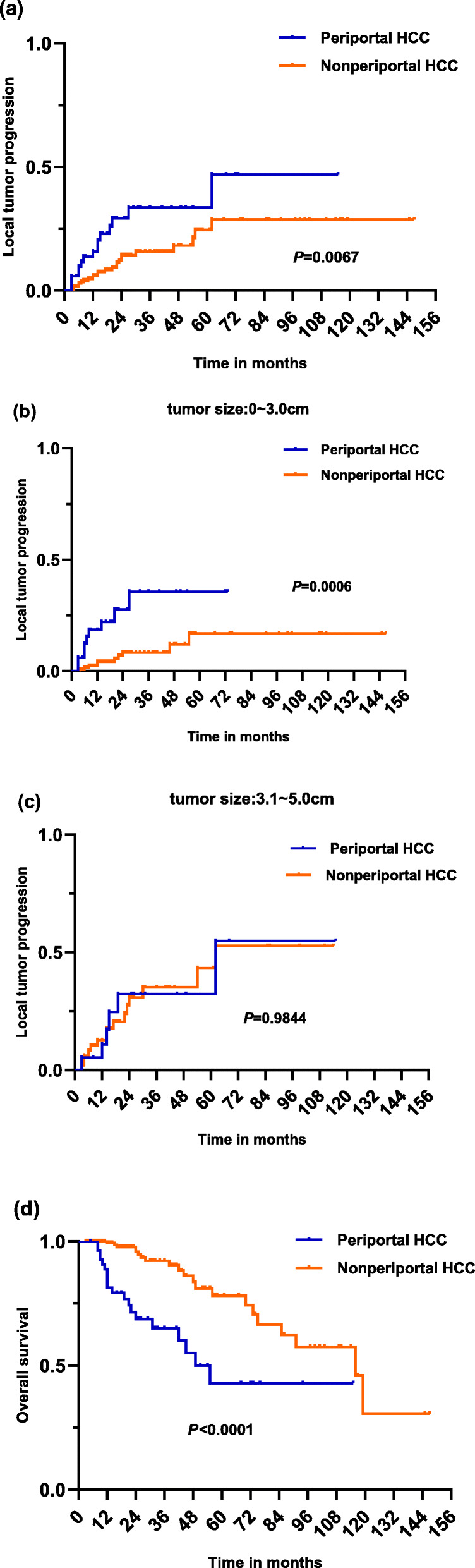
Kaplan–Meier curve demonstrating local tumor progression and overall survival of HCCs after RFA in the periportal and nonperiportal groups

The mean follow-up was 31 ± 24 months (range, 5.0–115.0 mo.) in the periportal group, and 33 ± 29 months (range, 3.0–145.0 mo.) in the nonperiportal group. Four patients in the periportal group were excluded to evaluate the LTP due to technical failure. Therefore, a total of 52 patients were analyzed. LTP occurred in 15 (28.8%) of 52 patients in the periportal group and 24 (13.6%) of 177 patients in the nonperiportal group(*P* = 0.0099). The LTP rates at 1, 3, and 5 years were significantly higher in the periportal group than those in the nonperiportal group (15.7, 33.7, and 46.9% vs 6.0, 15.7, and 28.7%; Fig. 2a: *P* = 0.007). In the subgroup of single HCC ≤ 3 cm, the LTP rates were significantly higher in the periportal group than those in the nonperiportal group (Fig.2b;*P* = 0.0006);however, there were no significant difference in LTP rate between two groups in the subgroup of single HCC of 3.1–5.0 cm in diameter (Fig.2c; *P* = 0.9844). In addition, we also observed the significantly worse DFS rates in the periportal group than those in the nonperiportal group (Supplemental Fig.1a-c). IDR was observed in 23 (44.2%) of 52 patients in the periportal group and 40 (22.6%) of 177 patients in the nonperiportal group(*P* = 0.0012). Extrahepatic recurrence was observed in 12 of 52 (23.1%) patients in the periportal group (range, 8–56 months; median, 23 months) and 14 of 177 (7.9%) patients in the nonperiportal group (range, 14–92 months; median, 34.5 months) (*P* = 0.0010). Portal vein tumor thrombus (PVTT) was observed in 16 (28.6%) of 56 patients in the periportal group (range, 1–46 months; median, 11 months) and 12 (6.8%) of 177 patients in the nonperiportal group (range, 5–75 months; median, 22 months) (P<0.0001). The univariate and multivariate analyses revealed that tumor size (HR [95%]: 1.553[1.151–2.095], *P* = 0.004), periportal HCC (HR [95%]: 2.200[1.111–4.358], *P* = 0.024) and AFP ≥ 400 (HR [95%]: 2.431[1.000–5.907], P = 0.004) were independent prognostic factors for LTP after RFA (Table 2). Salvage treatments for initial tumor recurrence or progression were shown in Table 3.

**Table 2:** Prognostic Factors for Local Tumor Progression (LTP) after RFA

Factor	All patients without LTP (*N* = 189, %)	All patients with LTP (*N* = 40, %)	Univariable Analysis		Multivariable Analysis	
			Hazard Ratio(95%CI)	*P* value	Hazard Ratio(95%CI)	*P* value
Child-Pugh class						0.783
A	174 (92.1)	36 (90.0)	1 (Reference)	0.512	1(Reference)	
B	15 (7.9)	4 (10.0)	1.416 (0.501–4.004)		1.163 (0.397–3.408)	
Etiology of tumor						
Hepatitis B virus	148 (78.3)	28 (70.0)	0.682 (0.238–1.953)	0.476	0.703 (0.228–2.170)	0.540
Hepatitis C virus	16 (8.5)	5 (12.5)	1.214 (0.324–4.548)		1.145 (0.271–4.834)	0.853
Non-B, non-C hepatitis	8 (4.2)	3 (7.5)	1.513 (0.337–6.795)	0.774	3.141 (0.626–15.749)	0.164
No	17 (9.0)	4 (10.0)	1 (Reference)	0.589	1 (Reference)	
AFP (ng/ml)						
≤ 20	115 (60.8)	23 (57.5)	1 (Reference)		1 (Reference)	
>20 and<400	55 (29.1)	8 (20.0)	0.881 (0.394–1.970)	0.757	1.133 (0.490–2.616)	0.770
≥ 400	19 (10.1)	9 (22.5)	2.466 (1.090–5.576)	0.030	2.431 (1.000–5.907)	0.050
Tumor size (cm)	2.67 ± 1.13	3.24 ± 1.10	1.529 (1.172–1.994)	0.002	1.553 (1.151–2.095)	0.004
TACE before RFA						0.770
Yes	122 (64.6)	28 (70.0)	1.216 (0.616–2.401)	0.573	0.898 (0.436–1.849)	
No	67 (35.4)	12 (30.0)	1 (Reference)		1 (Reference)	
Classification of HCC						0.024
Periportal HCC	37 (19.6)	15 (37.5)	2.370 (1.242–4.521)	0.009	2.200 (1.111–4.358)	
Nonperiportal HCC	152 (80.4)	25 (62.5)	1 (Reference)		1 (Reference)

**Table 3:** Events during the follow-up

Classification of all events	Periportal HCC (*N* = 56)	Nonperiportal HCC (*N* = 177)	*P* value
Tumor Events after RFA			
LTP	*15 (28.8%)	24 (13.6%)	0.0099
IDR	*23 (44.2%)	40 (22.6%)	0.0012
Extrahepatic recurrence	*12 (23.1%)	14 (7.9%)	0.0010
PVTT	16 (28.6%)	12 (6.8%)	<0.0001
2nd-line treatments			
TACE	1	3	–
RFA	6	11	–
TACE+RFA	11	18	–
Surgical resection	3	4	–
Liver transplantation	1	1	–
Radiation therapy	1	0	–
Systemic therapy	1	0	–
Systemic therapy+TIPS	1	0	–
Systemic therapy+TACE±RFA	5	9	–
Support therapy	3	5	–
Unknown	2	0	–
Major complication			0.1346
Total	6 (10.7%)	9 (5.1%)	
Hepatic infraction	2	0	
Portal vein thrombosis	4	5	
Biloma	0	1	
Liver abscess	0	1	
Bleeding	0	2	

*Four patients in the periportal group were excluded to evaluate the LTP, IDR and extrahepatic metastasis due to technical failure of RFA

### Overall survival and prognostic factors of tumor progression

The 1-, 3- and 5-year OS rates of periportal group were significantly better than those of nonperiportal group (81.3, 65.1 and 42.9% vs 99.3, 90.4 and 78.1%; Fig.2d: *P*<0.0001). In the subgroup of single HCC ≤3 cm, the OS rates were significantly poorer in the periportal group than those in the nonperiportal group (Supplemental Fig.2d;*P*<0.0001); And, a similar trend was observed in the subgroup of single HCC of 3.1–5.0 cm in diameter (Supplemental Fig.2e; *P* = 0.0584). The univariate and multivariate analyses revealed that tumor size (HR [95%]: 1.592[1.313–1.930], *P*<0.001), periportal HCC (HR [95%]: 2.417[1.559–3.745], *P*<0.001) and AFP ≥ 400 (HR [95%]: 2.955[1.652–5.283], *P*<0.001) were independent prognostic factors for tumor progression after RFA (Table 4). The median post-recurrence survival was 13 months in the periportal HCC group and 17.5 months in the nonperiportal group.

**Table 4:** Prognostic Factors for Tumor Progression* after RFA

Factor	All patients without tumor progression (*N* = 136, %)	All patients with tumor progression (*N* = 97, %)	Univariable Analysis		Multivariable Analysis	
			Hazard Ratio(95%CI)	*P* value	Hazard Ratio(95%CI)	*P* value
Child-Pugh classification						0.443
A	127 (93.4)	86 (88.7)	1 (Reference)	0.148	1 (Reference)	
B	9 (6.6)	11 (11.3)	1.593 (0.847–2.993)		1.290 (0.673–2.473)	
Etiology of tumor						
Hepatitis B virus	102 (75.0)	77 (79.4)	1.395 (0.607–3.205)	0.433	1.383 (0.574–3.330)	0.470
Hepatitis C virus	11 (8.1)	10 (10.3)	1.888 (0.683–5.213)		2.329 (0.786–6.899)	0.127
Non-B, non-C hepatitis	8 (5.9)	4 (4.1)	1.372 (0.385–4.881)	0.220	2.945 (0.784–11.057)	0.110
No	15 (11.0)	6 (6.2)	1 (Reference)	0.626	1 (Reference)	
AFP (ng/ml)						
≤ 20	88 (64.7)	52 (53.6)	1 (Reference)		1 (Reference)	
>20 and<400	37 (27.2)	26 (26.8)	1.387 (0.801–2.233)	0.179	1.720 (1.045–2.832)	0.033
≥ 400	11 (8.1)	19 (19.6)	3.289 (1.907–5.670)	<0.001	2.955 (1.652–5.283)	<0.001
Tumor size (cm)	2.40 ± 1.03	3.13 ± 1.13	1.518 (1.276–1.806)	<0.001	1.592 (1.313–1.930)	<0.001
TACE before RFA						
Yes	87 (64.0)	64 (66.0)	1.056 (0.693–1.610)	0.800	0.780 (0.498–1.220)	0.277
No	49 (36.0)	33 (34.0)	1 (Reference)		1 (Reference)	
Classification of HCC						
Periportal HCC	22 (16.2)	63 (64.9)	2.470 (1.622–3.761)	<	2.417 (1.559–3.745)	<
Nonperiportal HCC	114 (83.8)	34 (35.1)	1 (Reference)	0.001	1 (Reference)	0.001

*Tumor progression was defined as the appearance of any enhancing tumor foci in the liver or extrahepatic metastasis during the follow-up after ablation, including LTP, IDR, vascular invasion, extrahepatic metastasis

### Complications

There were no procedure-related mortalities in either group. Major complications occurred in 6 patients (10.7%) in the periportal group and 9 patients (5.1%) in the nonperiportal group, including two cases of hepatic infarction and four cases of portal vein thrombosis in the periportal group and one case of biloma, one case of liver abscess, two cases of bleeding and five cases of portal vein thrombosis in the nonperiportal group (Table 3). All complications were successfully treated.

## Discussion

In this study, we found that several newly recognized patterns of HCC recurrence or progression after RFA occurred frequently in patients with periportal HCC compared with those patients with nonperiportal HCC, such as rapid intrahepatic or extrahepatic progression and rapid portal vein tumor thrombosis, even the local complete ablation was achieved after the initial treatment. And these patterns of HCC recurrence or progression was strongly associated with periportal HCC, and indicated poor prognosis. More importantly, we also demonstrated that patients with single periportal HCC had worse local tumor control and survival than patients with single nonperiportal HCC in the treatment of RFA as the first-line therapy. This study therefore raises an important question about exploring the optimal ablation modality for single periportal HCC in the future studies.

**Fig. 3:**
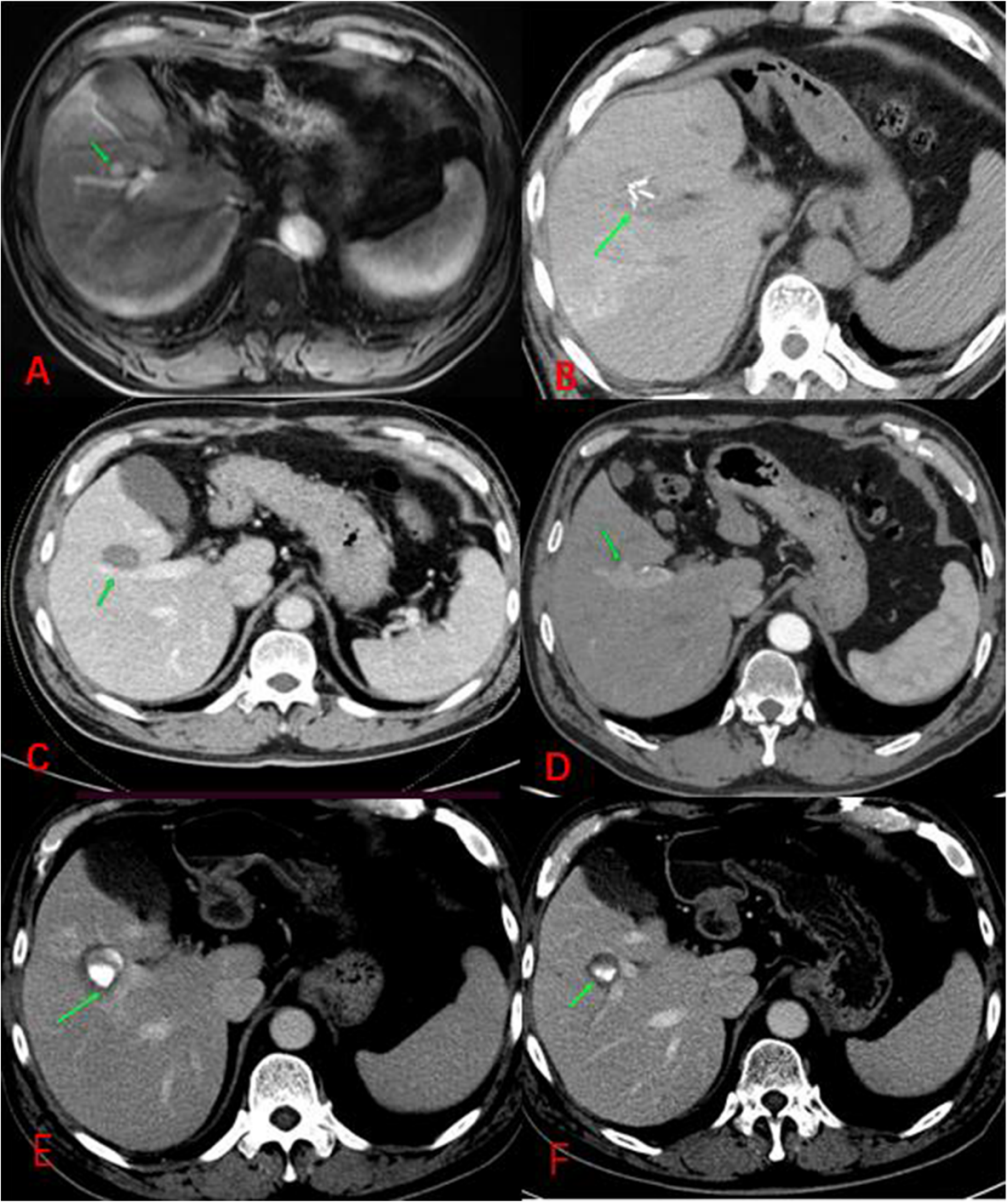
Local tumor progression (LTP) after RFA for periportal HCC in a 57-year-old man. (A) Dynamic contrast-enhanced axial magnetic resonance (DCE-MRI) scan obtained showing a small HCC (arrow) in periportal location before RFA. (B) CT scan obtained during RFA showing a multitip expandable electrode adjacent to the portal vein (arrow). (C) CT scan obtained during portal venous phase 1 months after RFA showing the complete ablation zone (arrow) adjacent to the portal vein. (D) CT scan obtained during hepatic arterial phase and portal venous phase (not shown) 53 months after RFA showing the LTP, a small arterial enhancing nodule (arrows), with washout at portal venous phase. (E) CT scan obtained during portal venous phase 1 months after TACE+RFA showing the complete ablation zone (arrow) adjacent to the portal vein, “intratumoral lipiodol deposition” can be seen in the ablation zone. (F) CT scan obtained during portal venous phase 4 months after TACE+RFA showing the complete ablation zone (arrow) adjacent to the portal vein

In terms of the technique of sufficient ablation, a safe ablation margin of 3 mm or more has been recognized as essential for preventing local tumor recurrence [15, 16]. However, it is practically difficult for periportal HCC to successfully create a safe ablative margin in the direction of the margin of HCC adjacent to the portal vein. In this study, periportal HCC after RFA exhibited a higher LTP rate than nonperiportal HCC. This was attributed to factors related to the adverse effect of the portal vein on RFA. First, the safe ablation margin was limited by anatomical location and the “heat sink effect” caused by the portal vein. Second, the adverse effect of the portal vein may reduce thermal damage to cancer cells, leading to viable residual cancer cells regenerating again during the follow-up (Fig. 3). However, the results of Kang et al. suggested that the long-term therapeutic outcomes of RFA for small perivascular HCC were similar to those for nonperivascular HCC [13]. This controversy may be attributed to several reasons. First, “perivascular” in their study was referred to as “periportal” and “perivenous”, but we only referred to periportal HCC. Second, the tumor sizes of periportal HCC in our study were larger. In addition, technical factors could lead to differences in these results, such as ablation strategy, radiofrequency power settings and operator experience. A recent study suggested that periportal HCC was an independent risk factor for LTP but not perivenous HCC, although the cause was unclear [8], and they refined “periportal HCC” instead of “perivascular HCC”.

**Fig. 4:**
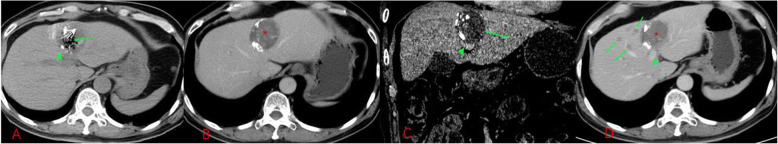
IDR with aggressive progression after RFA for periportal HCC in a 65-year-old man. (A) CT scan obtained during RFA showing a periportal HCC mass treated with TACE, and gasification (green arrowhead) was observed in the ablation zone during RFA. (B-C) CT scan obtained during the portal venous phase 1 month after RFA showing the complete ablation zone (arrow) adjacent to the portal vein (green arrowhead). (D) CT scan obtained during the portal venous phase 3 months after RFA showing multiple newly occurring small HCCs of similar size (arrow) surrounding the complete ablation zone (*)

**Fig. 5:**
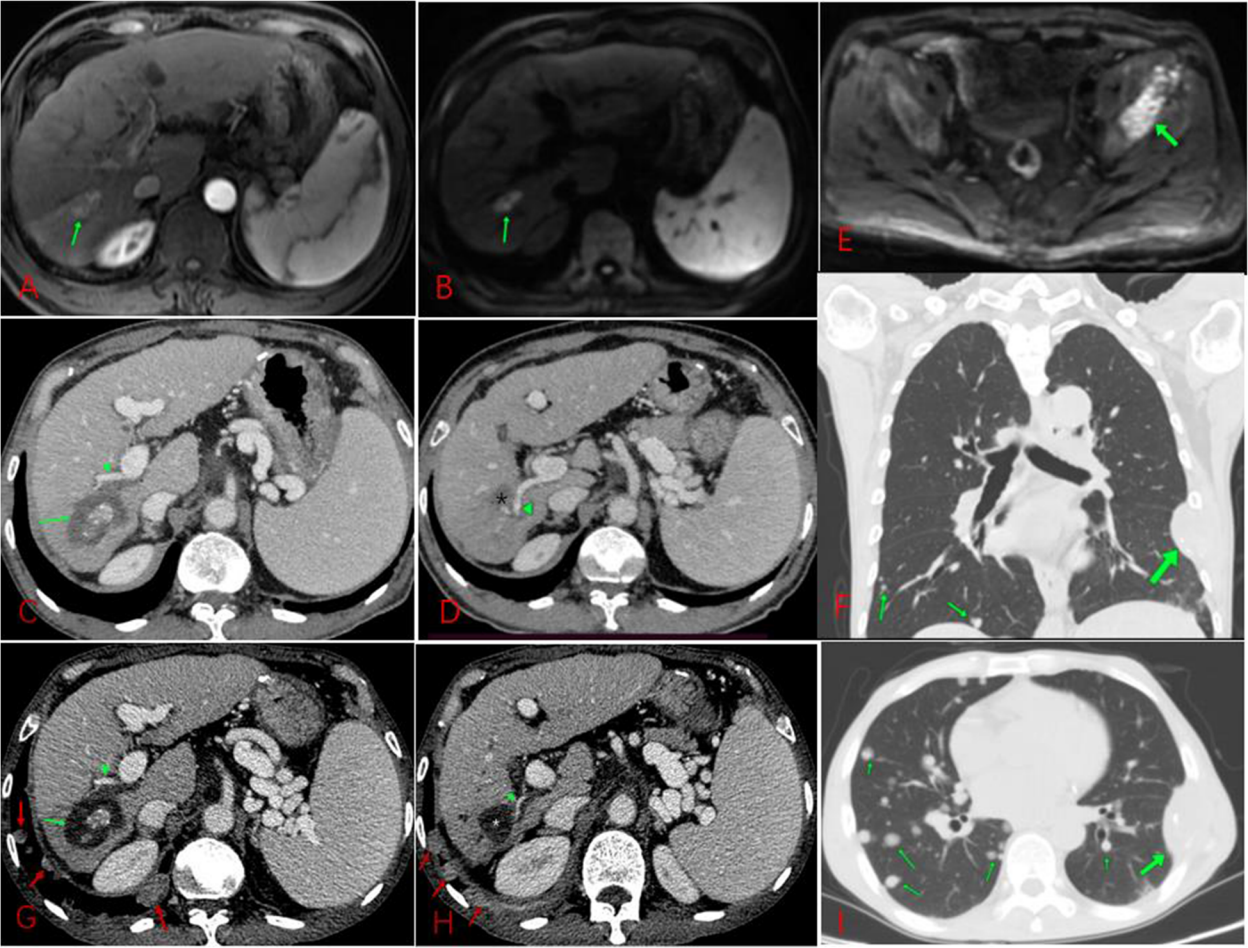
Extrahepatic recurrence with rapid progression after TACE+RFA for periportal HCC in a 61-year-old man. (A-B) DCE-MRI: hepatic arterial phase (A) and DWI (B) showing a small HCC (arrow) in the periportal location before TACE+RFA. (C-D) CT scan obtained during the portal venous phase 3 months after TACE+RFA showing the complete ablation zone (arrow) adjacent to the portal vein (arrowhead). (E) DWI scan obtained 6 months after TACE+RFA shows bone metastasis (arrow). (F) CT scan obtained 6 months after TACE+RFA showing thoracic wall metastases and multiple lung metastases (arrow). (G-I) CT scan obtained during the portal venous phase 12 months after TACE+RFA showing the complete ablation zone (arrow) adjacent to the portal vein accompanied by thoracic wall and lung metastases

To create a sufficient ablation zone for periportal HCC, portal vein injury seems to be inevitable due to puncture and thermal damage during RFA. However, the procedure of ablation for periportal HCC could increase the risk of rapid intrahepatic neoplastic progression, PVTT or extrahepatic spread through the injured portal system [9, 17]. Similar patterns of tumor recurrence associated with periportal HCCs have been reported in previous studies [10, 11, 18–20]. Among them, Kang et al. found that the rate of aggressive intrasegmental recurrence (AIR) was only 3.7% (20 of 539 patients) in all patients; however, the rate of AIR markedly increased to 15% (11 of 72 patients) in patients with periportal HCC [11]. Song et al. found that the rate of AIR was as low as 4.5% (1 of 22 patients) when TACE combined with RFA was used to treat periportal HCC [18]. In this study, three of 56 patients (5.4%) in the periportal group experienced IDR with aggressive progression after RFA (Fig. 4). Moreover, one patient (1.8%) with periportal HCC exhibited extrahepatic recurrence with rapid progression after RFA (Fig. 5); and another two patients (3.6%) with periportal HCC suffered RFA-related rapid PVTT within 7 months after RFA (Supplemental Fig. 2). All these patients showed complete ablation during the follow-up period, and there was no evidence of preablation extrahepatic metastasis or postablation intrahepatic recurrence. Among those new patterns of tumor recurrence or progression in this study, gasification in the ablation zone or even in portal vein during RFA was observed in most of those cases, which means increased intertumoral pressure during RFA due to the rapidly increased temperature. Therefore, gasification in the ablation zone or portal vein during RFA for periportal HCC may indicate a high risk of tumor recurrence or metastasis after ablation. Although the mechanism of those new patterns of recurrence is unknown, we suspect that rapid heating of periportal HCC during RFA and poorly differentiated HCC phenotype could play important roles in the process of tumor progression or recurrence after RFA; on the one hand, a suddenly increased intratumoral pressure due to rapid heating could promote viable tumor cells to spread directly into the peripheral liver or extrahepatic organs through the injured portal system [9, 11, 19]; On the other hand, poorly differentiated HCC phenotype may potentially increase the risk of tumor cells escape and distant metastasis during ablation [21, 22].

**Fig. 6:**
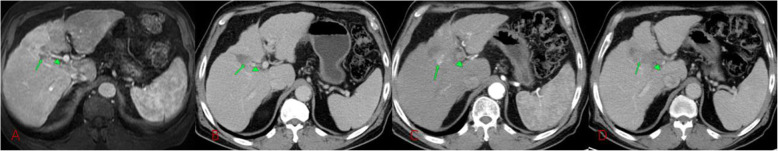
Residual tumor with rapid progression was observed in a 57-year-old man with periportal HCC, which showed direct invasion of the portal vein by residual tumor after RFA. (A) Dynamic contrast-enhanced axial magnetic resonance (DCE-MRI) scan obtained showing a small HCC (arrow) directly connected to the portal vein (arrowhead). (B) CT scan obtained during the portal venous phase 4 months after RFA showing the insufficient ablation margin (arrow) connected to the portal vein (arrowhead). (C)-(D) CT scan obtained during the hepatic arterial phase and portal venous phase 9 months after RFA showing local tumor progression accompanied by portal vein invasion (arrowhead)

In addition, residual tumor cells after RFA for periportal HCC can accelerate portal vein invasion. In contrast to nonperiportal HCC, due to the shorter distance that the residual cancer surrounded the ablation zone from the portal vein and the lack of a normal liver tissue barrier between them, the residual tumor cells tended to accelerate the process of PVTT. In this study, residual tumors directly resulted in portal vein invasion in two patients (3.6%) after initial RFA (Fig. 6). We also found that a higher incidence of PVTT with a shorter median time was observed in patients with periportal HCC than patients with nonperiportal HCC.

In this study, we identified three adverse risk factors (periportal, tumor size, and AFP ≥ 400 ng/ml) that predict tumor progression after RFA in the univariate and multivariate analysis. Interestingly, we also found that patients with single periportal HCC of 3-5 cm in diameter tended to have worse prognosis but similar local tumor control than those patients in the subgroup of nonperiportal HCC. This may be attributed to that tumor size is the most important factor of LTP, while “periportal” is one of the most important risk factors tumor progression and impairs long-term survival. In any case, particular caution must be taken in the treatment of periportal HCC with larger tumor size or AFP ≥ 400 ng/ml for ablation. However, there are no available guidelines for ablation for periportal HCC, and we have not been able to draw any conclusion about the efficacy of RFA using a multitip expandable electrode in the treatment of periportal HCC due to lack of sufficient evidence. Recently, a retrospective study with a small sample suggested that microwave ablation provides better local tumor control over RFA as a first-line therapeutic option for small single periportal HCC [23]. Further prospective controlled studies are urgently necessary to determine which ablation option among RFA, microwave ablation, cryoablation, and other ablation is best option for periportal HCC.

To reduce the potential risk of tumor spread during ablation, we recommend several ablation strategies in the treatment of periportal HCC for ablation based on our experiences, such as using a mono-tip electrode needle, managing longer ablation times at lower power to minimize gasification during ablation [11, 20], and combining RFA with TACE [18, 24]. Immediate imaging assessment after ablation and closer follow-up monitoring are necessary for patients with periportal HCC to prevent rapid tumor progression due to residual tumor or tumor recurrence. In addition, non-RFA-related technologies could provide an alternative option for periportal HCC to prevent or minimize newly recognized RFA-related tumor recurrence [20], such as microwave ablation [25], cryoablation [26] and irreversible electroporation [27], and iodine-125 seed implantation [28].

Our study has some limitations. First, this was a retrospective study with a small sample size based on medical records. Second, the exact cause of the new RFA-related tumor recurrence of periportal HCC lacked histopathological evidence, although the recurrence patterns did not occur in nonperiportal HCC. Third, data regarding the time interval between TACE and RFA were not evaluated. Finally, there was no uniform posttreatment or surveillance schedule.

## Conclusions

The long-term therapeutic outcomes of RFA as the first-line therapy for single periportal HCC were worse than those for single nonperiportal HCC, an increased higher risk of tumor recurrence or progression after RFA was significantly associated with periportal HCC. This study also suggested that tumor size, periportal HCC and AFP ≥ 400 were independent prognostic factors for tumor progression after RFA. This study therefore calls for further studies to explore the optimal ablation modality for single periportal HCC.

## Supplementary Information

**Additional file 1: Supplemental figure 1.** The Kaplan–Meier curve demonstrating the cumulative disease-free survival and overall survival of HCCs after RFA in the periportal and nonperiportal groups. **Supplemental figure 2**. Portal vein tumor thrombosis (PVTT) after RFA for periportal HCC in a 60-year-old woman. (A) CT scan obtained during hepatic arterial phase and portal venous phase (not shown) showing a periportal HCC accompanied by arterioportal fistula (arrowhead) that an arterial enhancing mass (arrows), with washout at portal venous phase. (B) CT scan obtained during RFA shows a periportal HCC mass treated by TACE, and portal vein (arrowhead) was observed during RFA. (C)-(D) CT scan obtained during hepatic arterial phase and portal venous phase 1 months after RFA showing the complete ablation zone (arrow) adjacent to the portal vein (arrowhead). (E)-(F) CT scan obtained during hepatic arterial phase and portal venous phase 7 months after RFA showing the complete ablation zone (*) adjacent to portal vein tumor thrombosis (arrowhead).

## Data Availability

The datasets used and/or analyzed during the current study are available from the corresponding author on reasonable request.

## Abbreviations

AFP: a-fetoprotein
AIR: Aggressive intrasegmental recurrence
BCLC: Barcelona clinic liver cancer
CT: Computed tomography
CBCT: Cone-beam computed tomography
HCC: Hepatocellular carcinoma
IDR: Intrahepatic distant recurrence
LTP: Local tumor progression
PVTT: Portal vein tumor thrombus
RFA: Radiofrequency ablation
TACE: Transarterial chemoembolization
