# Supplementary material for: Long-term outcome of percutaneous radiofrequency ablation for periportal hepatocellular carcinoma: tumor recurrence or progression, survival and clinical significance
Source: Cancer Imaging. 2022 Jan 4;22:2. doi: 10.1186/s40644-021-00442-2 (PMC8725335; doi:10.1186/s40644-021-00442-2)
Supplement: Supplementary file 1 — Additional file 1: Supplemental figure 1. The Kaplan–Meier curve demonstrating the cumulative disease-free survival and overall survival of HCCs after RFA in the periportal and nonperiportal groups. Supplemental figure 2. Portal vein tumor thrombosis (PVTT) after RFA for periportal HCC in a 60-year-old woman. (A) CT scan obtained during hepatic arterial phase and portal venous phase (not shown) showing a periportal HCC accompanied by arterioportal fistula (arrowhead) that an arterial enhancing mass (arrows), with washout at portal venous phase. (B) CT scan obtained during RFA shows a periportal HCC mass treated by TACE, and portal vein (arrowhead) was observed during RFA. (C)-(D) CT scan obtained during hepatic arterial phase and portal venous phase 1 months after RFA showing the complete ablation zone (arrow) adjacent to the portal vein (arrowhead). (E)-(F) CT scan obtained during hepatic arterial phase and portal venous phase 7 months after RFA showing the complete ablation zone (*) adjacent to portal vein tumor thrombosis (arrowhead). [file 40644_2021_442_MOESM1_ESM.docx]

**Supplemental figure 1.** The Kaplan–Meier curve demonstrating the cumulative disease-free survival and overall survival of HCCs after RFA in the periportal and nonperiportal groups.


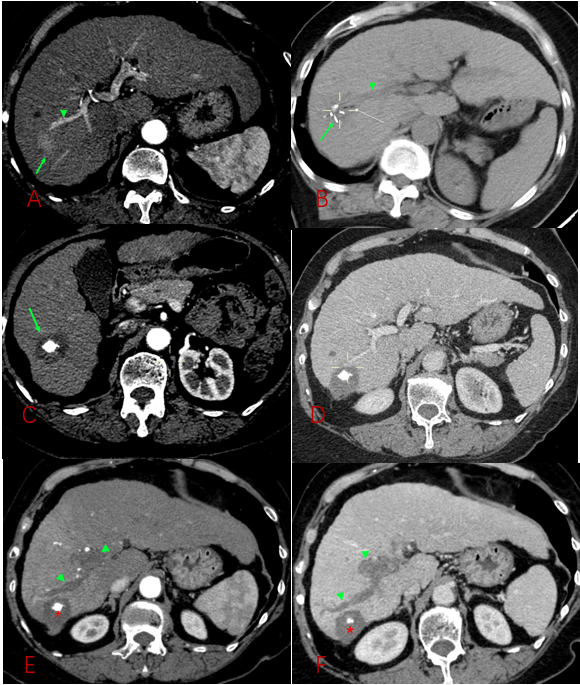


**Supplemental figure 2**. Portal vein tumor thrombosis (PVTT) after RFA for periportal HCC in a 60-year-old woman. (A) CT scan obtained during hepatic arterial phase and portal venous phase (not shown) showing a periportal HCC accompanied by arterioportal fistula (arrowhead) that an arterial enhancing mass (arrows), with washout at portal venous phase. (B) CT scan obtained during RFA shows a periportal HCC mass treated by TACE, and portal vein (arrowhead) was observed during RFA. (C)-(D) CT scan obtained during hepatic arterial phase and portal venous phase 1 months after RFA showing the complete ablation zone (arrow) adjacent to the portal vein (arrowhead). (E)-(F) CT scan obtained during hepatic arterial phase and portal venous phase 7 months after RFA showing the complete ablation zone (*) adjacent to portal vein tumor thrombosis (arrowhead).
